# Paired-pulse transcranial magnetic stimulation reveals probability-dependent changes in functional connectivity between right inferior frontal cortex and primary motor cortex during go/no-go performance

**DOI:** 10.3389/fnhum.2013.00736

**Published:** 2013-11-12

**Authors:** A. Dilene van Campen, Franz-Xaver Neubert, Wery P. M. van den Wildenberg, K. Richard Ridderinkhof, Rogier B. Mars

**Affiliations:** ^1^Department of Psychology, University of AmsterdamAmsterdam, Netherlands; ^2^Amsterdam Brain and Cognition, University of AmsterdamAmsterdam, Netherlands; ^3^Department of Experimental Psychology, University of OxfordOxford, UK

**Keywords:** rIFC, go/no-go task, paired-pulse, TMS, motor cortex, prediction, inhibition

## Abstract

The functional role of the right inferior frontal cortex (rIFC) in mediating human behavior is the subject of ongoing debate. Activation of the rIFC has been associated with both response inhibition and with signaling action adaptation demands resulting from unpredicted events. The goal of this study is to investigate the role of rIFC by combining a go/no-go paradigm with paired-pulse transcranial magnetic stimulation (ppTMS) over rIFC and the primary motor cortex (M1) to probe the functional connectivity between these brain areas. Participants performed a go/no-go task with 20% or 80% of the trials requiring response inhibition (no-go trials) in a classic and a reversed version of the task, respectively. Responses were slower to infrequent compared to frequent go trials, while commission errors were more prevalent to infrequent compared to frequent no-go trials. We hypothesized that if rIFC is involved primarily in response inhibition, then rIFC should exert an inhibitory influence over M1 on no-go (inhibition) trials regardless of no-go probability. If, by contrast, rIFC has a role on unexpected trials other than just response inhibition then rIFC should influence M1 on infrequent trials regardless of response demands. We observed that rIFC suppressed M1 excitability during frequent no-go trials, but not during infrequent no-go trials, suggesting that the role of rIFC in response inhibition is context dependent rather than generic. Importantly, rIFC was found to facilitate M1 excitability on all low frequent trials, irrespective of whether the infrequent event involved response inhibition, a finding more in line with a predictive coding framework of cognitive control.

## INTRODUCTION

A changing environment requires us to constantly adapt our behavior in response to new and surprising events. Suppressing unwanted actions or switching between response alternatives are important mechanisms to adapt our behavior. From a psychological perspective the set of mechanisms responsible for this flexible behavior are frequently grouped together under the umbrella term “cognitive control” (Miller, 2000; Ridderinkhof et al., 2004; Rushworth et al., 2004; Verguts and Notebaert, 2009). Response inhibition, i.e., the suppression of response activation of the upcoming action, is traditionally seen as one hallmark of cognitive control (Logan et al., 1984; Verbruggen and Logan, 2008; van den Wildenberg et al., 2010a). More recently, it has been argued that our brain implements control by employing a predictive strategy through which it extracts statistical regularities in the environment and uses this information to optimize response strategies (Friston, 2005; Clark, 2013). Evidence for predictive modulation of brain activity consistent with this model has been reported in parietal and frontal cortex (Huettel et al., 2005), but also in the primary motor cortex (Bestmann et al., 2008). In this framework, when an unpredicted stimulus occurs this results in a prediction error typically signaling adaptation of the predicted or planned motor response (“action reprogramming,” Mars et al., 2007b), and the updating of the internal representation of the environment (Mars et al., 2008; den Ouden et al., 2012).

A large body of literature has consistently identified a network of brain regions involved in cognitive control processes (Garavan et al., 1999; Ridderinkhof et al., 2004; Aron and Poldrack, 2006; Mars et al., 2007b, 2011). The right inferior frontal cortex (rIFC) in particular has been identified as a critical node within this network (for review see Ridderinkhof et al., 2011). Early studies suggested that this area is critical for the inhibition of inappropriate responses (Garavan et al., 1999; Aron et al., 2003; Rubia et al., 2003), thus specifying its role in action reprogramming and response inhibition. Consistent with this view, a series of studies recently showed that rIFC exerts an inhibitory influence over the primary motor cortex (M1) when actions need to be reprogrammed in the context of environmental information (Buch et al., 2010; Neubert et al., 2010). However, others have suggested a broader role for rIFC in action control. For instance, Verbruggen et al. (2010) suggested that different subparts of rIFC have distinct roles in detecting changes in the environment and implementing the most appropriate action. Vossel et al. (2011) showed that rIFC activity in response to unpredicted stimuli depends on the previous trial history, an interpretation consistent with a predictive coding framework of cognitive control such as discussed above.

Most current studies focusing on the role of rIFC in cognitive control present participants with an environment in which one stimulus-response combination is most frequent and occasional unexpected events require the dominant response to be overridden or replaced by an alternative action. These studies thus confound the requirement to inhibit a response and the surprise inherent in the unexpected stimulus. Thus, it cannot be fully established whether rIFC is involved primarily in response inhibition or more generally in the processing of unpredicted events. The goal of the current experiment is, therefore, to examine the influence of rIFC over the motor cortex in a context in which the role of response inhibition can be disentangled from a role in the processing of unpredicted events generally.

We employ a modified version of the classical “go/no-go” task. In this task, one type of stimulus is presented frequently while another type is presented infrequently. In the standard version of this task, participants are required to respond to the frequently presented stimulus and to withhold their action to the infrequently presented stimulus. In a modified version of the task we reversed the probabilities, such that the no-go stimuli are frequent and the go stimuli are unexpected. Thus, in this context, the unpredicted stimulus signals a need to override the pre-potent tendency to refrain from action (cf. Nieuwenhuis et al., 2003) and does not require response inhibition. Importantly this infrequent go stimulus is similar to the infrequent trials in the standard version of the task in terms of surprise. This set-up allows us to disentangle the role of IFC in response inhibition from a role in processing unexpected events in general.

To probe the influence of rIFC on the motor cortex, the functional connectivity between rIFC and M1 is assessed using paired-pulse transcranial magnetic stimulation (ppTMS). In this procedure, a single “test” transcranial magnetic stimulation (TMS) pulse is delivered over the hand representation of M1 to elicit a motor-evoked potential (MEP) in the electromygraphic (EMG) recorded from the effector muscle. On half of the trials, a “conditioning” TMS pulse over rIFC precedes the test pulse over M1. By calculating the ratio of the MEP amplitude recorded on paired-pulse (pp) and single-pulse trials, the impact of rIFC on the motor cortex can be assessed (Mars et al., 2009; Buch et al., 2010; Neubert et al., 2010; Buch et al., 2011; Catmur et al., 2011). Recent ppTMS studies showed rIFC exerts an inhibitory influence on M1 during action reprogramming (Buch et al., 2010; Neubert et al., 2010). This inhibitory effect was found when participants had to a switch between response alternatives. However, during normal action selection, a facilitatory effect of rIFC on M1 was reported.

The main aim of this study was thus to use physiological markers of the effects of rIFC on M1, as assessed by ppTMS, to disentangle the role of rIFC in response inhibition and signaling unpredicted actions in a go/no-go task. Manipulation of the probability of the no-go trials was used to differentiate between inhibitory demands *per se* from the action adaptation demands resulting from unpredicted action signals. In case rIFC is involved in response inhibition *per se*, we expected an influence of IFC on M1 on no-go trials only. Alternatively, in case of a more generic override-related activation of rIFC in response to unpredicted action signals in general, similar patterns are expected for infrequent no-go and infrequent go stimuli in both experiments.

## MATERIALS AND METHODS

### PARTICIPANTS

Eleven participants (age range 20–32 years, Mean 26.8 years SD 3.7, six women) performed experiment A (the *frequent go* experiment) and nine participants (age range 20–32 years, Mean 26.8 years SD 3.9, five women) performed experiment B (the *frequent no-go* experiment). Initially twelve participants were recruited for each experiment. One participant was excluded from the analyses of *frequent go* experiment, due to low trial numbers. One participant was excluded from the analyses of *frequent no-go* experiment, due to low trial numbers. Another two participants dropped out after *frequent go* experiment because the *frequent no-go* experiment could not be completed due to time restrictions. This resulted in a total of 11 participants in the *frequent go* (A) experiment and 9 participants in the *frequent no-go* experiment (8 of which participated in both experiments). All participants had normal or corrected-to-normal vision. All participants gave informed consent and were screened for familial epilepsy or other neurological disorders. A safety questionnaire was used to assess potential and risk factors of TMS. The Mid and South Buckinghamshire Research Ethics Committee approved the experimental procedures. At least 1 week before participating, participants were invited to a “taster” session, in which the whole procedure of the experiment was explained to them and they were given the opportunity to experience a few pulses of TMS, such that they could make an informed decision about their participation in the actual experiment. Participants participating in both experiments only attended the taster session before their first participation.

### EXPERIMENTAL SET UP

**FIGURE 1:**
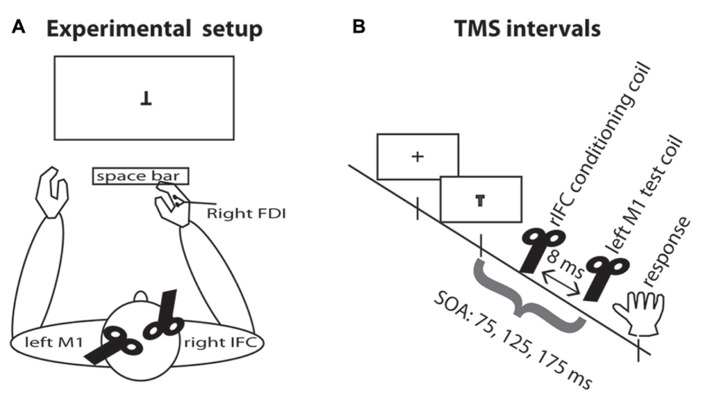
**Experimental setup.** **(A)** TMS setup and task display. Participants were seated in front of the computer display and responded by pressing the space bar with the right index finger. The test coil was placed over the left M1 and the conditioning coil was placed over rIFC. EMG was recorded continuously from the right hand FDI muscle. **(B)** TMS intervals. A fixation cross appeared on the screen for 500–750 ms. On TMS trials, a pulse of TMS over the motor cortex was applied on one of three SOAs, either 75, 125, or 175 ms. On half the TMS trials, this pulse was preceded by a pulse over rIFC 8 ms earlier.

All participants were seated approximately 85 cm in front of a computer screen and responded with the right index finger on the space bar (see **Figure 1**). A chin support system was used to prevent movement of the head during the experimental blocks. Participants wore earplugs to protect against the noise of TMS and an EEG cap on which the locations of the TMS stimulation sites were marked.

### DESIGN AND PROCEDURE

Stimuli were presented in white on a black background on a computer screen. A fixation cross was presented in the middle of the screen at the start of trial for 500–750 ms (uniform distribution) and disappeared at stimulus onset (SOA). A letter T (presented in regular orientation or upside-down) was used to represent go or no-go signals, respectively. This mapping was counterbalanced over participants. The stimuli disappeared after 60 ms.

When participants participated in both experiment A and B, the two experiments were conducted in two separate sessions on different days and separated by at least 1 week and the stimulus-response mapping were held constant across the two sessions for that participant. In experiment A, the “*frequent go*” experiment, participants were instructed to withhold their response on 20% of the trials (no-go trials) and respond as quickly as possible with the right hand by pressing the spacebar on 80% of the trials (go trials). In experiment B, the “*frequent no-go*” experiment, participants were instructed to withhold their response on 80% of the trials (no-go trials) and respond as quickly as possible on 20% of the trials (go trials).

Each experiment consisted of one behavioral practice block, a TMS practice block and eight experimental blocks of 120 trials each. The behavioral practice block and the TMS practice block consisted of 60 trials. In each experimental block, TMS was delivered on 24 trials on go and no-go trials, 12 single-pulse and 12 pp trials. TMS trials were mixed in a quasi-random fashion with no-TMS trials (either 4, 5, or 6 successive no-TMS trials in between TMS trials) to prevent expectancy of the TMS pulse. TMS pulses were delivered at one of three time intervals after SOA, namely 75, 125, or 175 ms. Overall, for each SOA there were 32 single-pulse trials and 32 paired-pulse trials (pp), resulting in a total of 192 TMS pulse trials distributed over go and no-go trials per experiment.

### TRANSCRANIAL MAGNETIC STIMULATION

Two figure-of-eight coils connected to MagStim 200 monopulse machines (The Magstim Company, Whitland, UK) were used to deliver TMS pulses. The TMS coil delivering the test pulse over left M1 was placed tangentially on the skull with the handle pointing backwards at an angle of approximately 45° (cf. O’Shea et al., 2007). The second coil delivering the conditioning pulse was placed initially over rIFC with the handle pointing forward. In a few participants the orientation of the coil was adjusted in a slightly counter-clockwise direction because the stimulation resulted in uncomfortable muscle contractions. The location of the coil over left M1 was determined as the location in which the largest MEP amplitude for any given stimulation intensity was elicited in the right first dorsal interosseous (FDI) muscle controlling the right index finger. The location of the conditioning coil was based on averaged MNI coordinates (*x* = 53, *y* = 15, *z* = 18), overlapping with previous work (Forstmann et al., 2008; Neubert et al., 2010; Verbruggen et al., 2010), transformed back into subject space for each individual and was placed using neuronavigation (MRI-aligned frameless stereotaxic neuronavigation, Brainsight, Rogue Research, Inc).

Resting motor threshold (RMT) in both left and right FDI were assessed, defined as the lowest intensity (expressed as % of maximum TMS stimulator output) at which an MEP with an amplitude of >50 μV is present in at least three out of five trials. The conditioning pulse intensity in the experiment was set at 110% of RMT of the right M1 elicited in the left FDI (cf. Mars et al., 2009; Buch et al., 2010). The intensity of the test pulse over left M1 was set at the value that yielded an average MEP of 1.0 mV recorded from the right FDI at rest. The inter-pulse interval for ppTMS was set at 8 ms. Mean RMT stimulator output over the right hemisphere was 35% for both experiments, established at the start of each experiment (range *frequent go* experiment: 28–44%; *frequent no-go *experiment: 30–40%). Mean left hemisphere stimulation intensity (1.0 mV) was 42% for both experiments (range *frequent go* experiment: 32–54%; *frequent no-go *experiment: 37–53%).

### ELECTROMYGRAPHIC RECORDINGS

Two Ag-AgCl electrodes were placed over the muscle belly of the FDI and the related muscle tendon on both the right and left hand. The left-hand electrodes were only used for determining the RMT and were removed before the start of the experimental blocks. An earth electrode was attached to the bony structure of the right elbow (olecranon ulnae). EMG data was sampled at 5000 Hz using a Cambridge Electronic Design (CED) 1902 amplifier, a CED Micro1401 Mk II. A/D converter. Bandpass filtering of 10–1000 Hz and using an additional 50 Hz notch filter was performed using Spike2 computer software (CED, Cambridge, UK). MEP amplitude was defined as the peak-to-peak amplitude within a window of 10–40 ms after the test pulse over M1.

### ANALYSES MEP DATA

Analyses of the MEP data followed standard procedures (Mars et al., 2007a, 2009). Trials were excluded if (a) the participant responded incorrectly (commission or omission errors), (b) the participant responded prematurely (RT < 50 ms), (c) no reliable MEP was elicited (MEP < 200 μV), (d) the MEP was elicited during or after the voluntary response, artificially inflating the MEP amplitude, or (e) there was a strong precontraction of the response muscle, again artificially inflating the MEP amplitude. Exclusion criteria (d, e) were based on trial by trial inspection and using a cut-off based of 10 root mean square EMG calculated during pre-stimulus baseline (100 ms prior to stimulus). To account for differences in overall MEP amplitude between participants, all MEP amplitudes were transformed into *z*-scores using all MEPs retained after preprocessing using the criteria outlined above of that participant over all conditions (Burle et al., 2002; van Campen et al., in press). To quantify the paired-pulse effect (PPE) of rIFC on M1, the ratio of MEP amplitudes between single-pulse and pp for each individual and SOA was indexed by *z*-scores per condition: PPE = (pp MEPmean - sp MEPmean) / [SD pp + sp], where pp is paired-pulse TMS and sp is single-pulse TMS (Buch et al., 2010). In this way a direct comparison between single-pulse and pp TMS is given.

### NORMALITY OF DATA

RT data were roughly normally distributed. In contrast, accuracy levels (i.e., error percentages) were not normally distributed. Therefore, analysis of variance (ANOVA) on error percentages were performed over square-root transformed percentages. Shapiro–Wilk tests for normality indicated that 3 out of 24 MEP samples (Experiment A: single-pulse TMS no-go trials at 75 ms, Experiment B: single-pulse and ppTMS go trials 175 ms) do not comply with the assumption of normality over participants. Because (1) the majority of the MEP samples is roughly normally distributed over participants, and (2) the ANOVA procedure is quite robust against moderate violations of the normality assumption (Schmider et al., 2010), we analyzed single-pulse and pp MEP amplitude with an overall omnibus mixed ANOVA. PPE data are normally distributed over participants, according to both Kolmogorov–Smirnov (*p* > 0.166) and Shapiro–Wilk (*p* > 0.183) tests of normality.

### ANALYSES

Mean RT and accuracy data were submitted to ANOVA with the between-subjects variable *Experiment* (*frequent go* vs. *frequent no-go*). An ANOVA with the within-subject factors *Trial-type *(*go *vs*. no-go trial*) and *SOA *(75, 125, and 175 ms) and the between-subjects factor *Experiment* (*frequent go* vs. *frequent no-go*) was used to analyze the single-pulse MEP data. An ANOVA with the within-subject factors *Pulse* (*single-pulse *vs.* paired-pulse*), *Trial-type *(*go *vs*. no-go trial*), and *SOA *(75, 125, and 175 ms) and the between-subjects factor *Experiment* (*frequent go* vs. *frequent no-go*) was used to analyze all MEP data. When the sphericity assumption was violated, degrees of freedom were corrected using the Greenhouse–Geisser (GG) method. Uncorrected degrees of freedom are reported for ease of reading.

It was not known at which time point in the response interval IFC exerted influence of the motor cortex. Therefore, we probed this interaction at the different SOAs. However, this also made our design unnecessarily conservative. Therefore, when looking at the specific effects on infrequent trials, which are the focus of the current manuscript, we followed hierarchical procedure to investigate these effects. First, we tested the PPE on infrequent trials for each SOA in each experiment against zero. Based on these analyses we identified that the 125 ms SOA in the *frequent go experiment* and the 175 ms SOA in the *frequent no-go experiment* were the moments at which IFC exerted its influence on M1. We then tested the differential effects of trial-type in the two experiments at only these SOAs in a single ANOVA. This procedure, though, does mean our data await replication in a separate experiment in which the SOAs are formulated in the hypothesis.

## RESULTS

Behavioral data are presented first, followed by the single-pulse MEP data, overall physiological data, and PPE analyses.

### BEHAVIORAL DATA

An ANOVA with the between-subjects factor *Experiment *(*frequent go* vs. *frequent no-go*) was used to analyze the RT and accuracy data. RTs on go trials were considerably faster in the *frequent go* experiment as compared to the *frequent no-go* experiment [333 vs. 431 ms, main effect *Experiment*, *F*_(1,1__8__)_ = 20.586, *p* < 0.001]. Fewer commission errors were made on frequent compared to infrequent no-go trials [0.3 vs. 20.8%, main effect *Experiment, F*_(1,18)_ = 45.251, *p* < 0.001]. Commission error responses were considerably faster on infrequent compared to frequent no-go trials [304 vs. 471 ms, main effect *Experiment*, *F*_(1,16)_ = 17.175, *p* = 0.001]. For go trials, omission error incidence was comparable across experiments [0.4 vs. 1.1%, *Experiment*, *F*_(1,18)_ = 0.533, *p* = 0.475]. These behavioral patterns were similar to a previous study using a similar probability manipulation (Nieuwenhuis et al., 2003).

### SINGLE-PULSE TMS

An ANOVA with the within-subject factors *Trial-type* (*go trials* vs. *no-go trials*), and *SOA* (75, 125, or 175 ms) and the between-subjects factor *Experiment* (*frequent go *vs.* frequent no-go*) was used to analyze the *z*-scored single-pulse MEP data. As expected MEP amplitudes were higher on go compared to no-go trials [0.042 vs. -0.101, main effect of *Trial-type*, *F*_(1,18) _= 6.111, *p* = 0.024]. Larger MEP amplitudes were found in the *frequent no-go* experiment [-0.088 vs. 0.029, main effect *Experiment*, *F*_(1,18)_ = 7.984, *p* = 0.011]. In addition, the difference in MEP amplitudes between go and no-go trials was modulated by the experimental context [interaction effect, *Trial-type* × *Experiment, F*_(1,18) _= 39.867, *p* < 0.001].

**FIGURE 2:**
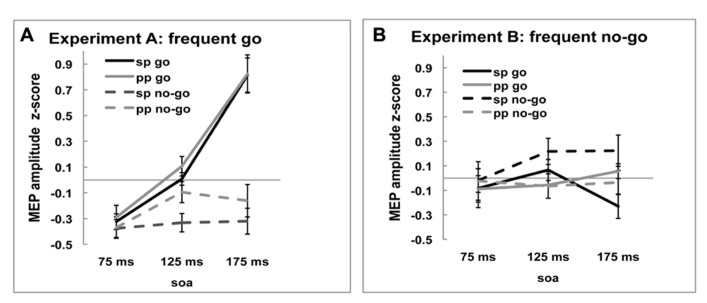
***Z*-scored MEP amplitudes of single-pulse (sp) and paired-pulse (pp) MEPs for go and no-go trials at three SOA for (A) *frequent go* experiment and **(B)***frequent no-go* experiment.** Black lines represent MEP amplitudes of single-pulse MEP amplitudes and gray lines of ppTMS MEP amplitudes. Solid lines are go trials and dotted lines are no-go trials.

Motor-evoked potential amplitudes differed depending on the time point of stimulation [-0.200, -0.010, and.122, main effect of *SOA*, *F*_(2,36)_ = 7.933, *p* = 0.001]. As can be seen in **Figure 2A**, in the *frequent go*, where responding to the stimulus was the predominant response and participants reacted fastest (see above, Behavioral results), the amplitude of the MEP increased over time when the stimulation occurred closer to the response. On the no-go trials, where participants were not required to make a response, this monotone increase is not observed. This pattern of go and no-go modulation of MEP amplitude was not seen in the *frequent no-go* experiment, presumably due to the much longer response times. These effects are reflected in the significant interactions, between *SOA* and *Experiment* [*F*_(2,36) _= 7.650, *p* = 0.002], between *SOA *and* Trial-type* [*F*_(2,36)_ = 3.677, *p* = 0.035] and between *Trial-type*, *SOA*, and *Experiment* [*F*_(2,36)_ = 16.963, *p* < 0.001].

In sum, the single-pulse MEP amplitudes during *frequent go* trials show the pattern normally observed during response trials and this pattern was modulated by the task manipulation with frequent no-go signals. These results suggest that our experimental manipulation was successful and hence we now turn to comparing the effects of rIFC stimulation on M1.

### PHYSIOLOGICAL EFFECTS OF ppTMS OVER rIFC ON M1

An ANOVA with the within-subject factors *Pulse* (single-pulse TMS vs. *ppTMS*), *Trial-type* (*go* vs. *no-go trials*), and *SOA* (*75*, *125*, or *175 ms*) and the between-subjects factor *Experiment* (*frequent go* vs. *frequent no-go*) was used to analyze the z-scored MEP data (**Figures 2A,B**). As was observed for the single-pulse data, MEP amplitudes on go trials were larger than on no-go trials [0.067 vs. -0.113, main effect of *Trial-type*, *F*_(1,18)_ = 16.103, *p* = 0.001], but this general pattern was different between the two experiments [*Trial-type* × *Experiment* interaction*, F*_(1,18) _= 40.469, *p* < 0.001]. There was no main effect of pulse [*Pulse*, *F*_(1,18)_ = 0.144, *p* = 0.709], but there were specific effects of *Pulse* between the two experiments [*Pulse* × *Experiment* interaction, *F*_(1,18)_ = 5.200, *p* = 0.035]. In the *frequent go* experiment, MEP amplitudes were lower following single-pulse TMS than ppTMS, whereas in the *frequent no-go experiment* this pattern was reversed. This was also reflected in the different trial-types [*Trial-type* × *Pulse* × *Experiment* interaction, *F*_(1,18)_ = 4.446, *p* = 0.049]. Thus, the presence of a pulse over rIFC affected the amplitude of the MEP elicited by the test coil in the two experiments differently.

Investigating the SOA-specific effects, we again observed that the MEP amplitudes changed over time [-0.197, -0.019, and 0.146, main effect of *SOA*, *F*_(2,36)_ = 13.182, *p* < 0.001]. The pattern of MEP amplitudes over time was different between the two experiments [interaction effect, *SOA* × *Experiment*, *F*_(2,36) _= 9.666, *p* < 0.001] and differed between go and no-go trials [interaction effect, *SOA* × *Trial-type*, *F*_(2,36)_ = 9.424, *p* = 0.002, GG-corrected: χ^2 ^= 9.757, ε = 0.696]. Also the pattern over time of MEP amplitudes on go and no-go trials was different between the two experiments [interaction effect, *Trial-type* × *SOA* × *Experiment*, *F*_(2,36)_ = 15.459, *p* < 0.001].

In summary, the effects of *Pulse* indicate that preceding the test pulse over M1 by a conditioning pulse over rIFC modulated the amplitude of the MEP. This effect is specific to *Trial-type* and *SOA*. Importantly, the reported effect differed between the two experiments. In the next section, we investigate these differences more closely using planned *t*-tests of the effects of the rIFC on M1 at each SOA and trial-type and within each experiment.

### PAIRED-PULSE EFFECTS: ALL SOAs

**FIGURE 3:**
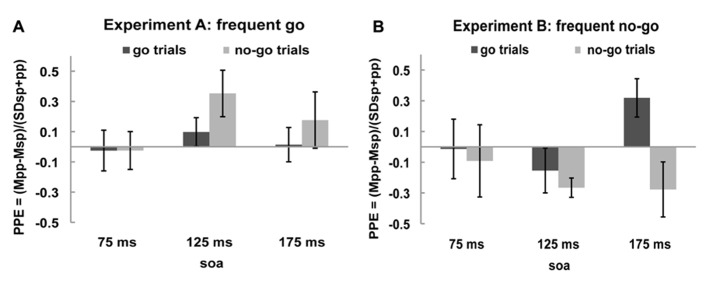
**Paired-pulse effect (PPE) no-go trials and go trials.** Black bars represent PPE of go trials and gray bars of no-go trials**for **(A)** *frequent go* experiment and **(B)** *frequent no-go* experiment.

In order to get a clearer picture of the effects of the rIFC pulse on the excitability of the motor cortex, we calculated the “PPE” for each time point and each condition (see Materials and Methods). The PPE has been established as a standard measure of causal influence of one cortical area over another (e.g., Civardi et al., 2001; Koch et al., 2006; O’Shea et al., 2007; Mars et al., 2009; Buch et al., 2010). If there is no effect of the rIFC pulse on the excitability of the motor cortex, i.e., on the MEP amplitude, PPE is zero. A positive PPE means that a pulse over rIFC increased the excitability of the motor cortex in that condition, termed “facilitation,” and a negative PPE indicates a decrease in M1 excitability, termed “inhibition.” These effects are displayed in **Figures 3A,B**.

One sample *t*-tests of the PPE against zero (the null hypothesis of no effect of rIFC stimulation) showed that in the *frequent go* experiment the only influence of rIFC on the motor cortex was a facilitatory effect on the infrequent no-go trials at the specific time interval of 125 ms after stimulus presentation [0.353, *t*_(10)_ = 2.298, *p* = 0.044, all other effects *p *> 0.30]. Interestingly, such a facilitatory effect was also present on the infrequent go trials in the *frequent no-go experiment*, with rIFC stimulation enhancing motor cortex excitability at the later time point of 175 ms [0.319, *t*_(8)_ = 2.560, *p* = 0.034]. Thus, there is a facilitatory effect of rIFC on M1 on infrequent trials, independent of their response (or inhibition) requirements. Inhibitory effects of rIFC on M1 were found in the *frequent no-go experiment* on no-go trials at 125 ms [-0.266, *t*_(8)_ = -4.230, *p* = 0.003]. A direct contrast of the facilitatory effect on infrequent no-go trials in the first experiment (frequent go trials) and frequent no-go trials in the second experiment revealed a significant difference between the two at SOA 125 ms [0.353 vs. -0.266, *t*_(18)_ = 3.729, *p* = 0.002, corrected for unequal variance]. No other effects reached significance (all *p *> 0.1).

### PAIRED-PULSE EFFECTS: EFFECTS OF LOW PROBABILITY

The goal of the experiment was to test whether the influence of IFC on M1 differed as a function of frequency between trial-types and experiments. Specifically, we were interested to see what would happen on low-frequency trials if they were traditional no-go trials or go trials. In order to ensure that we were able to detect any effects present, we probed the influence of IFC on M1 at three different SOAs. Thus far, we have reported effects while looking at all these SOAs. This design is, however, unnecessarily conservative, since we probe time points outside those on which we expect our effect. Therefore, we now present a more focused analysis, comparing the PPE on low frequent trials between the two experiment at the SOA that IFC influence is strongest. This SOA was defined for each experiment as the moment where the PPE of the low-frequent trial-types was maximum as indicated by the *t*-test of the PPE against zero. This was the 125 ms SOA for the *frequent go* experiment and the 175 ms SOA for the *frequent no-go* experiment.

We performed an ANOVA on the PPE on both go and no-go trials at those time points of maximum influence of IFC on M1. This ANOVA with within-subject factor *Trial-type* (*go trials* vs. *no-go trials*) and the between-subjects factor *Experiment* (*frequent go* vs. *frequent no-go*) for infrequent stimuli (SOA 125 and 175 ms) revealed an interaction between *Trial-type* and *Experiment* [*Trial-type* × *Experiment* interaction, *F*_(1,18)_ = 10.076, *p* = 0.005]. No main effect of *Experiment* or *Trial-type* was found [*Experiment, F*_(1,18) _= 1.958, *p* = 0.179, *Trial-type*, *F*_(1,18)_ = 1.616, *p* = 0.220]. This analysis thus shows that IFC influences M1 on different trial-types in the two experiments.

In conclusion, slower RTs on go trials and fewer commission errors on no-go trials were found in the *frequent no-go experiment* replicating previous results (Nieuwenhuis et al., 2003). Overall, differences in MEP amplitudes between the experiments were found indicating a different pattern of activation. Most important, facilitatory PPEs were observed not only on infrequent no-go trials, but also on infrequent go trials. The time difference between these PPEs (maximal at 125 and 175 ms after stimulus presentation, respectively) likely reflects the corresponding difference in response speed between the two contexts. In case of frequent no-go trials, an inhibitory PPE was found that peaked around 125 ms after stimulus presentation.

## DISCUSSION

The main goal of this study was to investigate the role of rIFC on the excitability of the primary motor cortex during cognitive control. Two main hypotheses can be formulated based on the literature, namely that rIFC functions to inhibit incorrect response tendencies or that rIFC responds to surprising stimuli in a more general sense. We manipulated the probability of no-go trials to differentiate inhibitory demands (withholding an action) from the action adaptation demands resulting from unpredicted events. Behaviorally, we observed faster responses on frequent as compared to infrequent go trials, and fewer commission errors on frequent as compared to infrequent no-go trials. Physiologically, facilitatory functional connectivity between rIFC and M1 was observed not only on infrequent no-go trials, but, crucially, also on infrequent go trials. This finding implies that rIFC influences M1 when unpredicted stimulus signals action adaptation demands in general, rather than just response inhibition *per se*.

The behavioral findings replicate previous work (Nieuwenhuis et al., 2003). These authors analyzed behavioral and event-related brain potential effects in a go/no-go task using frequent go and frequent no-go contexts. Physiologically, these authors first replicated typical findings, reporting a larger N2 event-related potential on infrequent no-go trials compared to frequent go trials, consistent with the then dominant account that N2 amplitude reflects inhibitory demands. However, when go- and no-go probabilities were reversed, they observed that the N2 disappeared for frequent no-go trials; instead, the N2 was now largest for the infrequent go trials, more consistent with an alternative account of the N2 in terms of conflict when the predicted action was to be overridden by a competing action option as designated by an infrequent signal. Their results are similar to the facilitatory PPE observed on the low frequent stimuli in the current experiments.

The physiological effects in the present study form an extension and in part an apparent departure from the results of previous PPE studies (Buch et al., 2010; Neubert et al., 2010). In the next section we will first contrast the current findings with previous studies using single-pulse TMS over M1 and secondly compare the current findings with other paradigms in which PPE is used as an index of functional connectivity. Finally, we will discuss the current findings within the existing framework of action control.

### SINGLE-PULSE EFFECTS

In this study, we exploited the use of TMS as a probe of cortico-spinal excitability. The amplitude of the MEP elicited by a single TMS pulse over the primary motor cortex can be taken as an index of the activity within cortico-spinal neurons (Civardi et al., 2001). This was clearly reflected in the development of the MEP on successive SOAs in the *frequent go *experiment. While MEP amplitudes were not significantly modulated during no-go trials, they increased during the stimulus-response interval on go trials. This is similar to effects standard observed in the literature (Leocani et al., 2000; Yamanaka et al., 2002; Coxon et al., 2006; van den Wildenberg et al., 2010b; Fujiyama et al., 2011). A different pattern was observed on *frequent no-go* experiment, which is partly explained by the longer response times in that experiment, resulting in the TMS pulses effectively occurring earlier in the response period. The pattern of MEP amplitudes during the go trials in the *frequent no-go* experiment might slightly surprising, showing a tendency to be a bit lower than baseline (75 and 125 ms) at SOA 175 ms, but it should be noted that it is not uncommon to see inhibitory processes within the motor cortex at work during longer stimulus-response intervals (Hasbroucq et al., 1999; Duque and Ivry, 2009). Importantly, in the current experiment, the single-pulse MEP amplitude is simply a baseline that is compared to the MEP amplitude on ppTMS trials. It is the modulation of the MEP amplitude by the preceding pulse over rIFC that is the dependent variable in this experiment.

### PAIRED-PULSE FUNCTIONAL CONNECTIVITY

In the current study we found facilitatory effects of rIFC on M1 on low frequent trials, independent of whether these trials required inhibition of a response. An inhibitory effect of rIFC on M1 was found only on frequent no-go trials. Previous work probing rIFC influence over M1 showed an inhibitory effect on switch trials around 175 ms and a facilitatory effect on stay trials (Neubert et al., 2010). This inhibition seemed to be preceded by a facilitatory effect of pre-SMA on M1 on action reprogramming trials (Mars et al., 2009; Neubert et al., 2010). One might expect action reprogramming trials and no-go trials to show similar dynamics, but this was not observed in the current experiment. Although the results of the present study make sense in the context of the literature on predictive coding and rIFC (e.g., Vossel et al., 2011), the results are not directly comparable with these previous ppTMS experiments. However a number of factors might reconcile these apparently different effects.

First, it seems likely that multiple processes occur in the time interval between stimulus and response. Rather than rIFC sending a single signal to M1 it seems more likely that there is a two-way stream of communication, with information going both from rIFC to M1 and back. It is known that rIFC interacts with M1 via both cortical and subcortical pathways (Neubert et al., 2010), the different functions of which are yet to be established. The current study is also the first to probe rIFC/M1 interactions during cognitive control outside the context of explicit action reprogramming and could therefore have tapped into previously unidentified interactions between the two cortical loci. Importantly, one should also be cautious relating *physiological* inhibition, as indicated by an inhibitory PPE, and *cognitive* inhibition, which is what is assumed to occur during response conflict resolution. Although the result from previous action reprogramming studies conveniently showed physiological inhibition where cognitive inhibition would be hypothesized, this inference warrants caution.

It should also be noted that the effects of ppTMS, although highly consistent and replicable between subjects and sessions (Neubert et al., 2010), are quite sensitive to even small changes in stimulation site and stimulation intensity (Civardi et al., 2001). Although we have kept the stimulation intensities in the current experiment to the same levels as in the previous experiments, the location of the rIFC coil might have been more ventral. In the study by Neubert et al. (2010), the coil ended up in location with a *z*-coordinate in MNI space of 25–30, whereas in the current study we aimed at a *z*-coordinate of 18. It is now becoming more and more apparent that the rIFC consists of a number of subdivisions, including different partitions in the dorsal-ventral dimension. It is also becoming clear that these subareas have different functions in cognitive control (Verbruggen et al., 2010). Therefore, it is not unlikely that our quite ventral simulation position targeted a different rIFC subdivision than the previous studies of Neubert et al. (2010) and Buch et al. (2010). This provides an interesting hypothesis for future studies.

### THE ROLE OF rIFC IN THE LARGER NETWORK

Cognitive control relies on a network of areas, prominently involving the rIFC, pre-supplementary motor area (pre-SMA), and basal ganglia (for reviews see Mostofsky and Simmonds, 2008; Mars et al., 2011; Ridderinkhof et al., 2011). It is therefore important to consider the present results in the context of this larger network, rather than presuming that rIFC functions alone to implement cognitive control. Work in primates (Isoda and Hikosaka, 2007) and humans (Nachev et al., 2007; Forstmann et al., 2008; Mars et al., 2009; Wylie et al., 2010) confirms an essential role not only for rIFC but also for pre-SMA and STN in conflict resolution. It has been shown that all nodes of this network are connected in the human brain (Aron et al., 2007) and that disruption of one node influences the activity and functional connectivity of the remaining nodes (Neubert et al., 2010).

The current results show a role of rIFC beyond response inhibition and it is worth considering what this means for its role in the larger network. Previous models in the interaction between the different nodes in this cognitive control network suggest that medial frontal cortex including pre-SMA is active before lateral frontal cortex including rIFC (Kouneiher et al., 2009; Neubert et al., 2010). The fact that rIFC is active on all types of surprising trials dovetails with similar observations of pre-SMA (Strange et al., 2005). These results invite an interpretation along the lines of predictive coding framework for the whole network and provide some possible clues on the function of rIFC within this network.

### INTERPRETATION LIMITATIONS

The current findings of IFC influence on M1 in response to infrequent stimuli provide evidence in line with the predictive coding account (Friston, 2005; Clark, 2013). However, some caution is warranted.

First, the exact timing of functional connectivity is difficult to predict. Therefore, we probed three well-documented time points (75, 125, and 175 ms), adding an additional factor to the design, making our design necessarily less powerful. Therefore, in the final part of the analyses, we used a series of *t*-tests to determine at which SOA the influence of IFC over M1 was largest on the infrequent trials. This turned out to be the 125 ms SOA in the frequent-go experiment and the 175 ms SOA in the *frequent no-go* experiment. We then performed a *Trial-type* × *Experiment *ANOVA on the PPE of these time points, showing clearly that the effect of rIFC on M1 is on different trial-types in the two experiments. In this way, we thus limit our interpretation to the trial-type and direction of the effect. We acknowledge that by analyzing the data in this fashion, we perform a delicate balance between the need to probe a range of time points in order to ensure we don’t miss detecting our effect and the need to not have a very underpowered design. The current results should thus be seen as preliminary. Future experiments should, for instance, perform these tests on different datasets, one focusing on identifying the SOAs that should be probed and a separate dataset to investigate the effect of frequency.

Second, we probed the functional connectivity between rIFC and M1 using an inter-pulse interval of 8 ms, which is similar to the timing of previous experiments (Buch et al., 2010; Catmur et al., 2011) and is thought to afford the involvement of direct cortical pathways (Neubert et al., 2010). Additional experiments employing longer inter-pulse intervals might reveal additional effects relying on sub-cortical pathways as well.

The current study thus invites a number of follow-up experiments to investigate top-down control over the motor cortex within a predictive coding framework, replicating the present effects in a large cohort and investigating the neural pathways mediating these effects. In general, we believe ppTMS has proven itself a suitable tool in this endeavor.

## Conflict of Interest Statement

The authors declare that the research was conducted in the absence of any commercial or financial relationships that could be construed as a potential conflict of interest.
